# Two Cases of Abdominal Surgery

**Published:** 1888-01

**Authors:** R. G. Bogue

**Affiliations:** Emeritus Professor of Surgery, Woman’s Medical College; 5 Washington Place


*TWO CASES OF ABDOMINIAL SURGERY.*

### TWO CASES OF ABDOMINAL SURGERY.

BY R. G. BOGUE, M. D.,

EMERITUS PROFESSOR OF SURGERY, WOMAN'S MEDICAL COLLEGE.

The patient, an unusually intelligent, unmarried woman, twenty-nine years of age, had suffered for five years almost constant distress, referred to the region of the ovaries. For a few days preceding and following the menstrual periods, the pain was severe; during the period of the menstrual hæmorrhage it was very severe, and during the five years mentioned, the patient was at no time entirely free from pain in the region of the ovaries. During the last four of these years she was confined to her room and bed. During all this time there was never any evidence of inflammation of the pelvic organs, and the ovaries were not appreciably enlarged. The following abridged account of her condition immediately prior to the operation is from a letter from her physician:

"She suffers almost constant nausea and dizziness and pain in the ovaries, which keep her nervous system in a highly disturbed condition. She seems to suffer a little more at each menstrual period than at the one preceding. Every measure for relief in such cases, known to me, has been tried and almost without effect. Any kind of an anodyne, even the local application of cocaine, intensifies the nausea to such a degree as to reduce her to the verge of starvation. She is, however, not hysterical, and there is no evidence of organic disease of the ovaries."

An exploratory incision two inches long was made in the median line of the abdomen at the junction of the lower and middle thirds of the distance from the umbilicus to the pubes.

The fallopian tubes and broad ligaments were intensely hyperæmic, but free from any inflammatory infiltration.

The surface of the ovaries was studded with small cysts, none of any considerable size, many being imbedded in the cortex of the organs.

The ovaries and two-thirds of each fallopian tube were removed. The broad ligament was transfixed by a needle armed with a double silk ligature, carbolized and waxed and the lateral halves tied separately in the usual manner. The abdominal wound was closed with six stitches of carbolized and waxed silk, and dressed with carbolized gauze.

With the exception of persistent nausea, the patient recovered from the anæsthetic and the operation promptly. Because of the inability of the stomach to retain food, nutrient enemata were given for a long time. She complained of pain in the pelvis, much as before the operation. The operation was followed by a severe and protracted attack of pelvic peritonitis and cellulitis, and an abscess finally, which pointed in the right iliac region. This, at first, seemed to be in connection with the stump of the broad ligament, but it closed so promptly after evacuation that it may have been only in the abdominal wall. She made a tedious recovery, and for a long time complained of tenderness on pressure over the pelvis, and the stomach continued very sensitive. There occurred quite an increase in pain and nervousness for a few days each time when the menstrual period would have occurred. When last seen she was much improved, and had commenced to gain in flesh.

II. Nephrectomy, in a case of pyonephrosis from a calculus in the pelvis of the kidney.

The patient, Mrs. M——, 32 years of age, married, and the mother of six children, in the fifth week after her last confinement, lifted and carried a heavy burden. This action was shortly followed by a feeling of stiffness and soreness in the right side.

Two months later she noticed a swelling in the right lumbar region, and during the following nine weeks was very ill. At this time, seventeen weeks after lifting the heavy weight, she was seen by Dr. Mary H. Thompson, of the Chicago Hospital for Women and Children, who found and opened a large abscess in the right lumbar region. This abscess cavity was freely washed out, and a drainage tube introduced. After this the discharge rapidly diminished to about one ounce of inodorous pus daily and the patient gained in flesh. This was her condition nine months after the original traumatism. About this time, through taking cold and domestic trouble, she suffered a relapse, and was brought to the hospital mentioned above, in September, eleven months after the original attack. When admitted she could not stand erect, suffered very severe pain in the right lumbar region, had no appetite, and was sleepless. The sinus was enlarged, the former treatment was repeated, and she improved for a time.

On October 6, pus was first found in the urine, and it steadily increased in quantity. There was great soreness in the right leg, which finally she was unable to extend. The inguinal glands became much swollen and very tender. During the first week in November her suffering was extreme. The highest temperature observed was 101.6° Fahr.

On November 11, about thirteen months after the first symptoms of the trouble were observed, the patient was anæsthetized, and the kidney removed. The incision was made midway between the crest of the ilium and the twelfth rib, parallel with the latter. The kidney was about double the size of a man's fist, and reached almost to the brim of the pelvis and to the umbilicus. The operation was tedious and protracted, because of the difficulty experienced in including the attachment in a ligature. This was caused mainly by the large size of the calculus in the pelvis of the organ, which prevented securing of the part with the forceps. Finally, a heavy ligature of carbolized and waxed silk was placed around the pedicle and securely tied. The kidney was then removed by dividing it into two portions, and each of them taken out separately. All bleeding vessels were secured by ligatures or by hæmostatic forceps. The cavity was cleansed with a two per centum carbolized solution, and a dressing of carbolized gauze applied. The calculus weighed 315 grains, and measured four inches in circumference in its longest diameter, and three and one-quarter inches in its shortest diameter.

The patient rallied fairly for a short time, and then sank and died two hours after the operation, from shock incident to the prolonged operation and loss of blood.

I am satisfied that in these cases, especially when the kidney is large, the best plan would be to freely open the kidney, preferably by means of the galvano- or thermo-cautery, thus avoiding hæmorrhage, since even a thin sac bleeds freely upon incision or laceration. The sac should then be allowed to drain freely and to shrink in size, until it can at some subsequent time be removed with less difficulty.

5 Washington Place.

